# Rehabilitation approach after 2 years of the COVID-19 pandemic: lessons to be learned

**DOI:** 10.31744/einstein_journal/2023CE0367

**Published:** 2023-04-18

**Authors:** Ricardo Kenji Nawa, Suelen Elaine Uhlig, Carla Luciana Batista, Raquel Afonso Caserta Eid, Caroline Gomes Mól

**Affiliations:** 1 Hospital Israelita Albert Einstein São Paulo SP Brazil Hospital Israelita Albert Einstein, São Paulo, SP, Brazil.

Dear Editor,

The coronavirus disease 2019 (COVID-19) pandemic has infected more than 623 million people globally, decimating over 6.55 million lives.^(1)^ In Brazil, the cumulative cases exceeded 34.77 million with more than 687,500 deaths.^(1)^ The high number of patients with severe acute respiratory coronavirus-2 (SARS-CoV-2) infection dramatically increased the rate of hospitalization and need for intensive care support, burdening health systems worldwide.^(2)^ As this was an unknown disease, guidelines and recommendations were gradually being published by medical organizations to provide knowledge and assist frontline multidisciplinary teams.

The recommendations for physiotherapists were first published in the early 2020; these focused on workforce planning and preparation, treatments, and personal protective equipment for physiotherapists to provide safe and adequate assistance during the acute phase of COVID-19.^(3)^ Additionally, an update for clinical practice recommendations was published in 2022, which included topics such as: workload management, staff health, including vaccination, clinical education, personal protective equipment, and interventions, including awake prone position, early mobilization, and rehabilitation of patients with hypoxemia.^(4)^ The purpose of screening hospitalized patients daily was to identify those with clinical stability (*e.g.,* hemodynamic, respiratory, neurological, and cardiovascular stability), and initiate rehabilitation interventions to prevent or minimize muscle and strength loss, thereby promoting functional recovery.^(4)^ Current literature suggests that muscle mass, strength, and physical function are highly interconnected.^(5)^ Therefore, prolonged periods of bed restriction and inactivity may greatly impact patients’ physical function.^(5)^ Most survivors of severe COVID-19 have experienced severe muscle wastage, persistent functional disabilities, and higher levels of dependency in daily-living activities even after hospital discharge. The use of standard metrics is highly recommended for evaluating patients’ muscle mass (*e.g.,* point-of-care ultrasound),^(5,6)^ muscle strength (*e.g.,* medical research council sum score),^(5)^ and physical function (*e.g.,* mobility and functional scales) to ensure that resources are used optimally and therapeutic interventions are planned effectively.^(5)^

Considering the most recent recommendations, a structured rehabilitation program for hospitalized patients with COVID-19 which includes standard metrics for screening and classifying patients’ rehabilitation stage can be an important strategy to adapt the therapeutic interventions to individual patients’ needs, especially in terms of the modality, time, dose, and intensity of the interventions.^(7)^The survivors of critically ill episodes may present secondary disabilities as a result of intensive care treatments, known as post-intensive care syndrome (PICS).^(8)^ Early rehabilitation and physiotherapy interventions are highly recommended to treat and prevent these conditions, and should continue after hospital discharge to improve the long-term recovery and functional independence of patients.^(8)^ Severe COVID-19 survivors who were assessed one year after admission into the ICU showed physical (74.3%), mental (26.2%), and cognitive (16.2%) symptoms.^(9)^ Even patients who did not require hospital admission had substantial long-term disabilities related to COVID-19 infection. Additionally, over half of the patients with severe COVID-19, reported experiencing fatigue and two-third reported new physical problems such as weakness, joint stiffness, joint pain, muscle weakness, myalgia, and dyspnea. The critical illness also resulted in many survivors having work-related problems.^(9)^ This scenario posed challenges for rehabilitation after hospital discharge, because in addition to the physical, mental, or cognitive symptoms that were frequently reported in COVID-19 survivors, “social distancing” was recommended.^(9,10)^ As a result of social distancing, the pandemic favored the use of technology such as videoconference and phone communication, as an alternative to rehabilitation services at a distance.^(11,12)^ In initial trials, exercise programs delivered by telerehabilitation showed efficiency in improving functional capacity, physical performance, and dyspnea in individuals with post-COVID conditions, although the certainty of evidence was low or very low.^(11)^
